# Impact of cardiovascular and immunologic variables on subclinical carotid atherosclerosis in subjects with anti-phospholipid antibodies

**DOI:** 10.1016/j.dib.2018.06.083

**Published:** 2018-06-27

**Authors:** Matteo Nicola Dario Di Minno, Giacomo Emmi, Pasquale Ambrosino, Antonella Scalera, Antonella Tufano, Giovanni Cafaro, Rosario Peluso, Alessandra Bettiol, Gerardo Di Scala, Elena Silvestri, Domenico Prisco

**Affiliations:** a Department of Translational Medical Sciences, Federico II University, Naples, Italy; b Department of Experimental and Clinical Medicine, University of Florence, Florence, Italy; c Department of Clinical Medicine and Surgery, Federico II University, Naples, Italy; d Department of Neurosciences, Psychology, Pharmacology and Child Health (NEUROFARBA), University of Florence, Florence, Italy

## Abstract

Whereas some previous data on carriers with isolated antiphospholipid antibodies positivity (APP) suggested an increased risk of arterial events in this clinical setting, no data are available on subclinical atherosclerosis in this clinical setting. This article reports data on intima-media thickness of the common carotid artery (CCA-IMT) and of the Bulb (Bulb-IMT) and on the prevalence of carotid plaques in APP carriers and in subjects with antiphospholipid syndrome (APS) specifically stratifying for the presence of thrombotic manifestations, cardiovascular risk factors, antibody isotype and concomitant Systemic Lupus Erythematosus (SLE) or other autoimmune diseases.

**Specifications Table**

**Table:** 

Subject area	Medicine
More specific subject area	Vascular medicine
Type of data	Common carotid artery intima-media thickness (CCA-IMT), intima-media thickness at the level of carotid Bulb (Bulb-IMT) and prevalence of carotid plaques in subjects with antiphospholipid syndrome (APS), in carriers with isolated antiphospholipid antibodies positivity (APP) and controls stratified for the presence of thrombotic manifestations and cardiovascular risk factors, antibody isotype and Systemic Lupus Erythematosus (SLE) or other autoimmune diseases
How data was acquired	Ultrasound machine (MyLab 25 Gold, Esaote, Florence, Italy) with a 7.5–12 MHz linear-array transducer.
Data format	Mean with standard deviations analyzed with t-test for continuous data and percentages analyzed with the χ2 test for categorical variables
Data source location	Federico II University, Naples, Italy

**Value of the data**

● We here report data on common carotid artery intima-media thickness (CCA-IMT), intima-media thickness at the level of carotid Bulb (Bulb-IMT) and prevalence of carotid plaques in subjects with antiphospholipid syndrome (APS) and in carriers with isolated antiphospholipid antibodies positivity (APP) stratifying for the type of thrombotic manifestations, for the presence of cardiovascular risk factors, antibody isotype and concomitant Systemic Lupus Erythematosus (SLE) or other autoimmune diseases.
● Cardiovascular and immunologic variables impact on subclinical atherosclerosis and data corrected for these potential confounders are needed.
● These data can be useful to be combined with data from other studies in the frame of a meta-analysis.

## 1 Data

Data on CCA-IMT, Bulb-IMT and on the prevalence of carotid plaques in APS subjects, in APP carriers and in matched controls stratified for the presence of thrombotic manifestations and cardiovascular risk factors, antibody isotype and Systemic Lupus Erythematosus (SLE) or other autoimmune diseases [1].

## 2 Experimental design, materials, and methods

As further detailed elsewhere (http://dx.doi.org.10.1016/j.ijcard.2018.06.010) consecutive subjects with a persistent positivity for antiphospholipid antibodies (lupus anticoagulant [LA], IgG and IgM anti-cardiolipin [aCL], IgG and IgM anti-β_2_ glycoprotein-I [aβ_2_GPI]) [2] were classified as APS in the presence of a history of an objectively documented venous and/or arterial thrombosis and/or with recurrent miscarriage or as APP when clinical history, physical examination and diagnostic procedures (electrocardiogram and vascular ultrasound) excluded the presence of symptomatic and/or asymptomatic venous and/or arterial thrombotic events and of recurrent miscarriages. Data about cardiovascular risk factors were recorded according to the National Cholesterol Education Program (NCEP) criteria [3]. An ultrasound assessment of carotid intima-media thickness (IMT) was performed as previously described [4] to evaluate CCA-IMT, Bulb-IMT and the presence of carotid plaques, defined as an IMT ≥ 1.3 mm.

Statistical analysis was performed with the IBM SPSS 22 system (SPSS Inc., Chicago, IL, USA). Continuous data were expressed as mean ± standard deviation (SD). The *t*-test was performed to compare continuous variables and categorical variables were expressed as % and analyzed with the χ^2^ test. When the minimum expected value was < 5, the Fisher׳s exact test was used. All the results are presented as 2-tailed values with statistical significance if *p* values < 0.05.

### 2.1 Effect of different types of thrombotic events on subclinical atherosclerosis

APS subjects with arterial thrombotic manifestations showed the highest CCA-IMT and Bulb-IMT values, and the highest prevalence of carotid plaques. The difference was significant both versus controls and versus APP carriers. In contrast, APS subjects with non-arterial thrombotic events (including venous thrombosis and recurrent miscarriage) showed a more severe atherosclerosis than controls, whereas no difference was found as compared to APP carriers (Table 1).

**Table 1:** Common Carotid Artery Intima-Media Thickness (CCA-IMT), intima-media thickness at the level of carotid Bulb (Bulb-IMT) and prevalence of carotid plaques in carriers of antiphospholipid antibodies positivity (APP), subjects with antiphospholipid syndrome (APS) and controls, stratified for the type of thrombotic manifestation.

	**CCA-IMT (mm)**	***P* vs Controls**	***P* vs APP**
**Controls**	0.82 ± 0.12	Comparator	0,032
**Arterial APS**	1.09 ± 0.68	< 0.001	< 0.001
**Non-arterial APS**	0.88 ± 0.29	0,035	1.000
**APP**	0.89 ± 0.25	0,032	Comparator
	**Bulb-IMT (mm)**	***P* vs Controls**	***P* vs APP**
**Controls**	0.95 ± 0.18	Comparator	0,009
**Arterial APS**	1.43 ± 0.89	< 0.001	< 0.001
**Non-arterial APS**	1.18 ± 0.56	< 0.001	0.807
**APP**	1.10 ± 0.45	0,009	Comparator
	**Carotid Plaques *n* (%)**	**P vs Controls**	**P vs APP**
**Controls**	33 (10.2%)	Comparator	< 0.001
**Arterial APS**	24 (49.0%)	< 0.001	0.077
**Non-arterial APS**	61 (35.5%)	< 0.001	0.795
**APP**	35 (33.7%)	< 0.001	Comparator

Note: Arterial APS includes cardiovascular and cerebrovascular events; Non-arterial APS includes venous thrombosis and recurrent miscarriage.

### 2.2 Effect of cardiovascular risk factors on subclinical atherosclerosis

To also evaluate whether traditional cardiovascular risk factors might impact on the difference in subclinical atherosclerosis found among APS subjects, APP carriers and controls, we repeated analyses only selecting subjects without any cardiovascular risk factor, and we found a significantly higher CCA-IMT and Bulb-IMT with a marginally significantly higher prevalence of carotid plaques both in APP carriers and in APS subjects, as compared with controls (Table 2).

**Table 2:** Common Carotid Artery Intima-Media Thickness (CCA-IMT), intima-media thickness at the level of carotid Bulb (Bulb-IMT) and prevalence of carotid plaques in carriers of antiphospholipid antibodies positivity (APP), subjects with antiphospholipid syndrome (APS) and controls. Sub-analysis in subjects without cardiovascular risk factors.

**Variable**	**Controls**	**APP**	**P vs controls**	**APS**	***P* vs controls**	***P* vs APP**
	**(*n* = 63)**	**(*n* = 29)**		**(*n* = 43)**		
**CCA-IMT (mm)**	0.71 ± 0.22	0.81 ± 0.19	0.037	0.85 ± 0.28	0.005	0.504
**Bulb-IMT (mm)**	0.83 ± 0.22	1.04 ± 0.46	0.007	1.09 ± 0.58	0.001	0.699
**Plaques *n* (%)**	4 (6.35%)	6 (20.7%)	0.051	10 (23.2%)	0.017	0.797

Plaques: defined as IMT ≥ 1.3 mm.

### 2.3 Effect of antiphospholipid antibody isotype on subclinical atherosclerosis

**Fig. 1:**
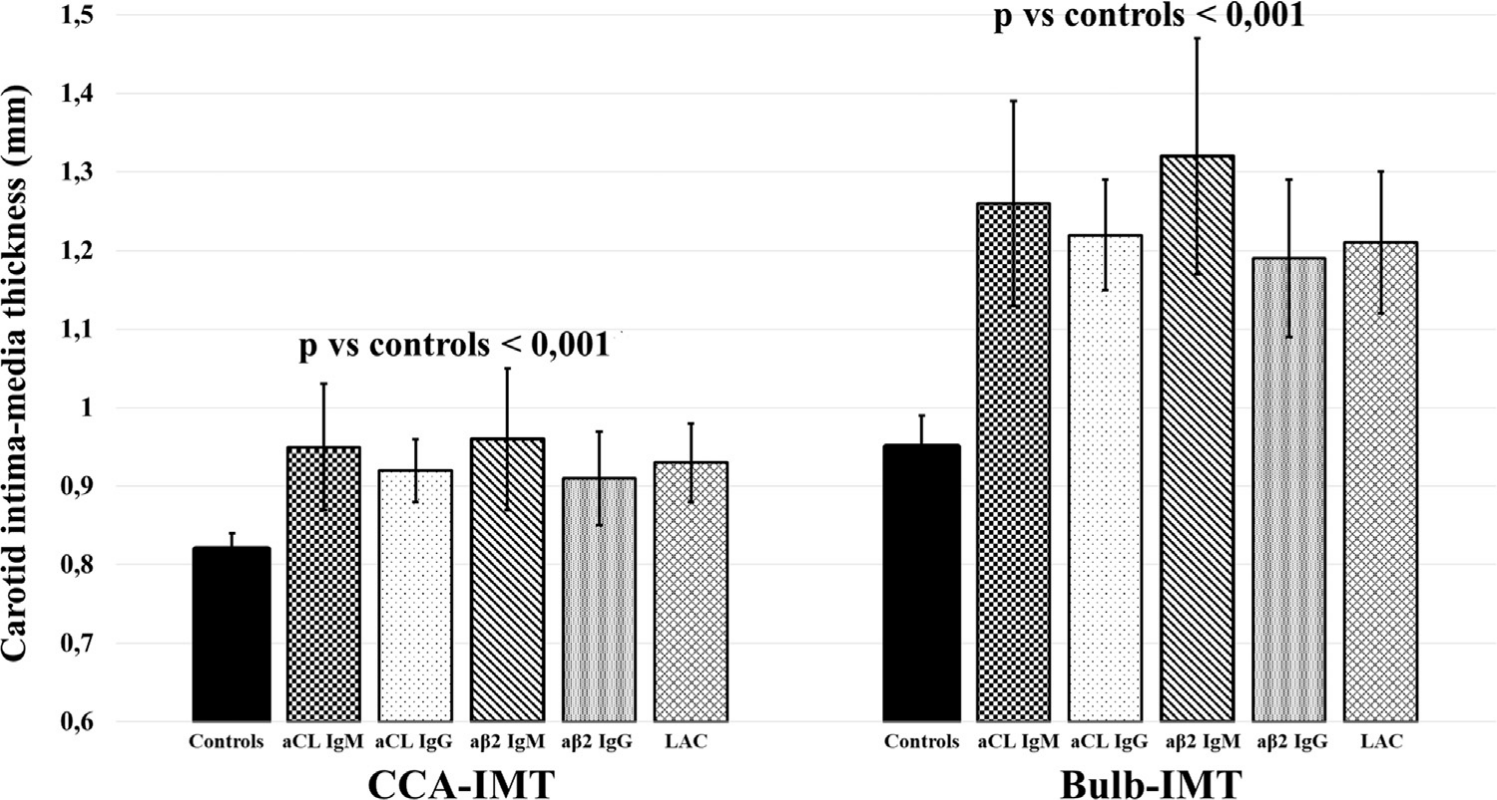
Common Carotid Artery Intima-Media Thickness (CCA-IMT), Intima-Media thickness at the level of carotid bulb (Bulb-IMT) in carriers of antiphospholipid antibodies positivity (APP), subjects with antiphospholipid syndrome (APS) and controls stratified according to the antiphospholipid antibody isotype.

Some evidence [5] suggested that IgG and IgM isotypes of antiphospholipid antibodies define different subsets of thrombo-embolic events with IgG being most prevalent in subjects with venous thrombosis, IgM being primarily found among patients with arterial events. Thus, we have evaluated CCA-IMT, Bulb-IMT and the prevalence of carotid plaques stratifying population according to the antiphospholipid antibody isotype (Fig. 1).

### 2.4 Effect of co-existing auto-immunity disorders on subclinical atherosclerosis

**Fig. 2:**
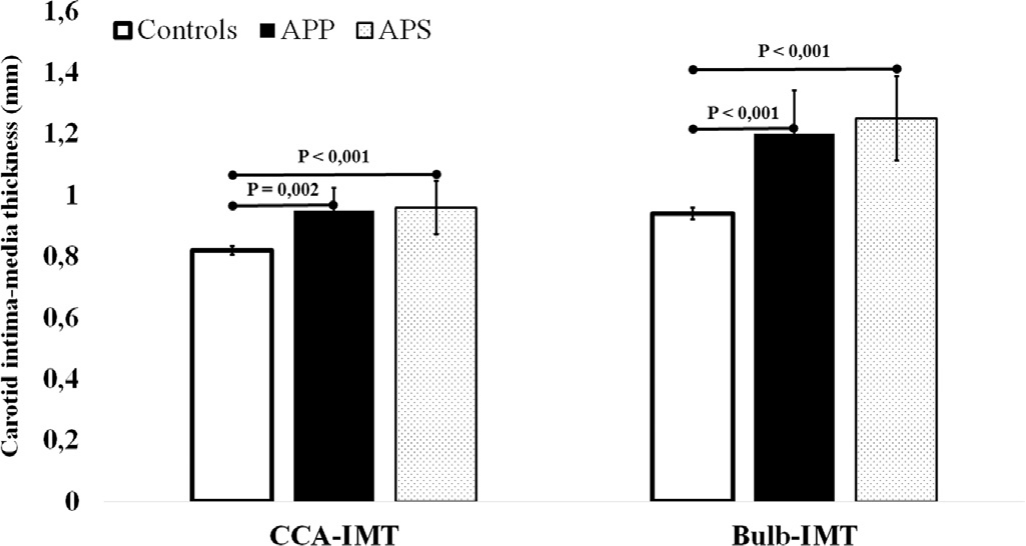
Common Carotid Artery Intima-Media Thickness (CCA-IMT) and Intima-Media thickness at the level of carotid bulb (Bulb-IMT) in carriers of antiphospholipid antibodies positivity (APP), subjects with antiphospholipid syndrome (APS) and controls after excluding subjects with positivity for Systemic Lupus Erythematosus or other autoimmune diseases.

To avoid the potential confounding effect of co-existing autoimmune diseases on subclinical atherosclerosis [6], all the analyses were repeated after excluding subjects with Systemic Lupus Erythematosus (SLE) or other autoimmune diseases and, as compared with controls, a higher CCA-IMT, Bulb-IMT and prevalence of carotid plaques were confirmed in APP carriers and in APS subjects (Fig. 2).
