# Supplementary material for: Impact of cardiovascular and immunologic variables on subclinical carotid atherosclerosis in subjects with anti-phospholipid antibodies
Source: Data Brief. 2018 Jun 27;19:1799–803. doi: 10.1016/j.dib.2018.06.083 (PMC6141369; doi:10.1016/j.dib.2018.06.083)
Supplement: Supplementary file 1 — Transparency document [file mmc1.docx]

Conflict of interest disclosure

All the Authors have nothing to declare
